# Comparison of linkage disequilibrium and haplotype diversity on macro- and microchromosomes in chicken

**DOI:** 10.1186/1471-2156-10-86

**Published:** 2009-12-20

**Authors:** Hendrik-Jan Megens, Richard PMA Crooijmans, John WM Bastiaansen, Hindrik HD Kerstens, Albart Coster, Ruud Jalving, Addie Vereijken, Pradeepa Silva, William M Muir, Hans H Cheng, Olivier Hanotte, Martien AM Groenen

**Affiliations:** 1 Animal Breeding & Genomics Centre, Wageningen University, Wageningen, The Netherlands; 2 Department of Animals, Science and Society, Faculty of Veterinary Medicine, Utrecht University, Utrecht, The Netherlands; 3 Hendrix Genetics, Boxmeer, The Netherlands; 4 Department of Animal Sciences, Faculty of Agriculture, University of Peradeniya, Sri Lanka; 5 Department of Animal Sciences, Purdue University, West Lafayette, IN, USA; 6 United States Department of Agriculture, Agricultural Research Service, Avian Disease and Oncology Laboratory, East Lansing, MI, USA; 7 University of Nottingham, Population and Evolutionary Genetics Dept. Nottingham, UK; 8 International Livestock Research Institute (ILRI), Nairobi, Kenya, Africa

## Abstract

**Background:**

The chicken (*Gallus gallus*), like most avian species, has a very distinct karyotype consisting of many micro- and a few macrochromosomes. While it is known that recombination frequencies are much higher for micro- as compared to macrochromosomes, there is limited information on differences in linkage disequilibrium (LD) and haplotype diversity between these two classes of chromosomes. In this study, LD and haplotype diversity were systematically characterized in 371 birds from eight chicken populations (commercial lines, fancy breeds, and red jungle fowl) across macro- and microchromosomes. To this end we sampled four regions of ~1 cM each on macrochromosomes (GGA1 and GGA2), and four 1.5 -2 cM regions on microchromosomes (GGA26 and GGA27) at a high density of 1 SNP every 2 kb (total of 889 SNPs).

**Results:**

At a similar physical distance, LD, haplotype homozygosity, haploblock structure, and haplotype sharing were all lower for the micro- as compared to the macrochromosomes. These differences were consistent across populations. Heterozygosity, genetic differentiation, and derived allele frequencies were also higher for the microchromosomes. Differences in LD, haplotype variation, and haplotype sharing between populations were largely in line with known demographic history of the commercial chicken. Despite very low levels of LD, as measured by r^2 ^for most populations, some haploblock structure was observed, particularly in the macrochromosomes, but the haploblock sizes were typically less than 10 kb.

**Conclusion:**

Differences in LD between micro- and macrochromosomes were almost completely explained by differences in recombination rate. Differences in haplotype diversity and haplotype sharing between micro- and macrochromosomes were explained by differences in recombination rate and genotype variation. Haploblock structure was consistent with demography of the chicken populations, and differences in recombination rates between micro- and macrochromosomes. The limited haploblock structure and LD suggests that future whole-genome marker assays will need 100+K SNPs to exploit haplotype information. Interpretation and transferability of genetic parameters will need to take into account the size of chromosomes in chicken, and, since most birds have microchromosomes, in other avian species as well.

## Background

Accurate characterization of linkage disequilibrium (LD) within and between populations is important in domestic animal genetic studies because LD underlies all forms of mapping studies [1] and is an important parameter in the design of marker panels. A recent study on LD in the chicken (*Gallus gallus*) [2] showed substantial differences in the extent of LD between populations, which is consistent with pronounced differences observed between breeds of other domesticated species [3,4]. The extent of LD in broilers estimated by SNP genotyping was shown to be very limited with r^2 ^values below 0.3, on average, for distances between 0.25 and 1 cM [2]. Studies in layers suggest higher extent of LD compared to broilers, although studies on LD in chicken other than broilers are sparse [5,6]).

Despite low levels of LD in broilers, Andreescu et al [5] showed that there is significant overlap in the LD of marker pairs between different populations. This result suggests that haplotypes are shared between commercial broiler lines. Estimating haplotype sharing between populations is of interest because it can aid in predicting transferability of genetic parameters, such as genomic estimated breeding values (GEBV) [7] or QTL, from one population to another. Characterization of LD and haplotype sharing has been achieved in humans by making extensive haplotype maps of different populations [8,9]. These maps confirmed the organization of haplotypes in so called haploblocks. The elucidation of genome-wide haploblock structure has been beneficial for designing SNP genotyping assays that capture the maximum amount of haplotype diversity in humans [10]. The design of a genome-wide haplotype map for chicken would be equally beneficial for the design of high-density genotyping assays.

In most birds, the genome is organized into a few very large chromosomes and many very small chromosomes[11,12]. The best studied bird genome to date, that of the chicken, has five so called macrochromosomes (GGA1-5) that range in size from 50 to 200 Mb, five intermediate chromosomes (GGA6-10) ranging from 20 to 40 Mb, and 28 microchromosomes (GGA11-38) that average ~12 Mb. The smallest of the microchromosomes were estimated to be less than 5 Mb[13].

The microchromosomes have structural differences compared to the larger chromosomes, such as higher GC content. Intergenic distances on the microchromosomes are also lower as well as the average size of the introns, resulting in a much higher gene density compared to the macrochromosomes [13]. Recombination rates in microchromosomes (50 - 100 kb/cM) are much higher compared to the macrochromosomes (~300 kb/cM), possibly resulting from the requirement of at least one chiasma per chromosome per meiosis, and possibly facilitated by a higher density of cohesin binding sites[14,15].

The higher recombination rate on microchromosomes is expected to reduce LD compared to macrochromosomes. This conclusion was ascertained by Aerts et al [2]for one microchromosome indicating that LD may not be the same throughout the chicken genome. However, a systematic survey of differences in haplotype structure between micro- and macrochromosomes in birds has not been done. Recombination rate was also shown to correlate positively with nucleotide diversity in chicken [16]. The degree of haplotype sharing is expected to decline with increasing recombination rate, which should result in lower transferability of genetic parameters for microchromosomes. Other measures such as haploblock structure, are also expected to be affected by differences in recombination rate. All SNP based studies so far have used SNP densities that were insufficient to ascertain LD and haplotype structure in chicken.

We applied a focused high-density SNP typing strategy, sampling 1 SNP every 2 kb, to quantify the differences in LD, haplotype diversity, haplotype sharing and haploblock structure between the micro- and macrochromosomes in chicken. For a good representation of both types of chromosomes, we sampled four regions of ~1 cM each on macrochromosomes (GGA1 and GGA2), and four 1.5 -2 cM regions on microchromosomes (GGA26 and GGA27). To test the generality of our conclusions, a broad and diverse set of populations were genotyped including all the important commercial types (white egg and brown egg layers, sire and dam broilers)[17], as well as two traditional Dutch fancy breeds and a wild chicken population.

## Methods

### Chicken populations

Commercial and non-commercial populations were surveyed to provide a broad view of LD patterns and haplotypic diversity in chicken. Unrelated individuals were taken from each population. For the commercial populations, representatives of all major types were selected: one white egg layer (E2), one brown egg layer (B2), two female or dam broiler lines (one of a closed line - E5, and one of an open line - A3), and one male or sire broiler line (E3). The commercial lines were provided by Hendrix Genetics; E2, E5 and E3 lines were surveyed previously [2]. Furthermore, two traditional Dutch breeds (Owl Bearded, AvDiv_09, and Frisian Fowl, AvDiv_10), and one wild chicken population (*Gallus gallus spadiceus*, AvDiv_101) were sampled; these populations were previously surveyed in the AvianDiv project [18]. We also included seven *Gallus lafayetii *(Ceylon Jungle Fowl, SRI) specimens, from Sri Lanka, for outgroup comparison and determining the ancestral state of the SNPs. Additional details on the populations and sampling can be found in Table 1.

**Table 1:** An overview of the chicken populations population

population label	type/population	origin	N indiv.	GGA1 H_obs_	GGA2 H_obs_	GGA26 H_obs_	GGA27 H_obs_	Anc. Freq.
E2	white egg layer	Hendrix Genetics	54	0.11	0.09	0.04	0.14	0.66
B2	brown egg layer	Hendrix Genetics	62	0.18	0.17	0.21	0.28	0.68
E3	male broiler	Hendrix Genetics	61	0.23	0.23	0.20	0.3	0.68
E5	female broiler (closed)	Hendrix Genetics	57	0.24	0.19	0.14	0.17	0.68
A3	female broiler (open)	Hendrix Genetics	58	0.24	0.25	0.25	0.28	0.68
AvDiv_101	Red Jungle Fowl *(G. g. spadiceus)*	Thailand	29	0.25	0.28	0.25	0.27	0.71
AvDiv_9	Owl Bearded	The Netherlands	26	0.21	0.24	0.27	0.22	0.65
AvDiv_10	Frisian Fowl	The Netherlands	24	0.20	0.21	0.25	0.28	0.66
	*G. lafayetii *(CJF)	Sri Lanka	7	0.02	0.03	0.03	0.04	1.00

An overview of the populations surveyed. Population label refers to the names of the same population in other studies: the wild chicken population, Frisian Fowl, and Owl Bearded were part of the AvianDiv project [18]. The E2, E5 and E3 lines have been previously studied by Aerts et al. (2007). Indicated are the number of specimens used (N indiv), observed heterozygosity per chromosome (H_obs_), and overall ancestral allele frequencies (anc. Freq.).

### SNP selection and typing

SNPs were selected from dbSNP 125 and mapped to *Gallus gallus *build 2.1. Four chromosomes, two macro- (GGA1 and GGA2) and two microchromosomes (GGA26 and GGA27) were each surveyed at two regions, for a total of eight regions. The size of each region was ~300 kb (~1 cM) for the macrochromosomes, and ~150 kb (~1.5-2 cM) for the microchromosomes [14,15]. Regions were selected for having sufficient SNP information to allow selection of one SNP per 2 kb and good reliability of the assembly. Furthermore, regions on the same chromosome were chosen to be far apart, to minimize effects of hitchhiking due to linkage. One SNP was selected per 2 kb, on average, with a total of 889 SNPs (Additional File 1).

Genotyping was performed using the GoldenGate/Sentrix Array technology from Illumina [19], according to manufacturer's protocols. The 889 SNPs were part of a larger 1536-plex assay.

### LD analysis

LD was calculated as pairwise r^2 ^and D' values using Haploview 4.0 [20] for each of the populations and for each of the genomic regions. For each population, only markers with a 75% or higher genotyping success, without significant deviation from Hardy-Weinberg (p < 0.001), and with a minor allele frequency (MAF) greater than 5% were included in the analysis.

Observed values of r^2 ^were fitted to the Sved equation ([21], see also [6,4]),

where *LD*_*ijk *_is the observed *LD *for marker pair *i *of population *j *in region *k*, *d*_*ijk *_is the distance in *bp *for marker pair *i *of population *j *in genomic region k, *β*_*jk *_is the coefficient that describes the decline of *LD *with distance for population *j *in genomic region *k *and *e*_*ijk *_is a random residual. For each genomic region within population  was estimated using the nls function in the R environment http://www.r-project.org/.

Population and genomic region effects on LD extent were tested using *LDc*_*ijk*_, which is the distance corrected and variance stabilized LD for marker pair *i *in genomic region *k *and breed *j *and it was estimated using  and  obtained with equation 1 [4]:

Differences in LD between genomic regions and populations were analyzed by testing their significance when included as fixed effect in a linear model [4].

Effective population sizes were estimated by transforming physical distances to genetic distances (300 kb/cM for the macrochromosomes, and 65 kb/cM for the microchromosomes [15]). Past effective population sizes were calculated using a sliding window and transforming physical distances to genetic distances to estimate the number of past generations as [22-24]

### General population statistics

Observed heterozygosity and MAF were calculated with custom Perl scripts. The ancestral state of the SNPs was determined by assessing the state of the SNP in the seven Ceylon Jungle Fowl samples, and the allele present in the same monomorphic state in all seven animals was inferred to be the ancestral state for *G. gallus*. Tests for the effect of genomic region on heterozygosity and on ancestral frequency were done using a linear model.

Genetic distances were calculated based on allele frequencies using the Gendist program, and a neighbor joining tree was constructed using the Neighbor program from the Phylip package [25].

### Haplotype and haploblock analysis

Haploview was used for inferring haplotypes and haplotype frequencies using the '-blocks' option [20], using custom Perl scripts to generate block definitions and to collect haplotypes and haplotype frequencies for a sliding window (scripts available upon request from HJM). Haplotype homozygosity (HH) was calculated as the sum of products of haplotype frequencies [26]. Haploblock structure was determined using two haploblock rules, the Gabriel rule [10] and the 4 gamete rule, as implemented in Haploview 4.0 [20], based on haplotypes with 5% or higher occurrence.

Haplotype sharing between two populations was calculated as the number of haplotypes that were shared by both populations divided by the average number of haplotypes in these populations. The average number of haplotypes was calculated by taking the sum of the haplotype count of both populations divided by two. Only haplotypes with greater than 5% occurrence in each of the populations were considered.

## Results

### SNP genotyping

Of 889 SNPs assayed, 806 were successfully genotyped. A 90% success rate is comparable to previous data sets generated for chicken on the same platform [27]. The numbers of SNPs successfully genotyped per region can be found in Additional File 1. Of these 806 SNPs, 91% had a MAF greater than 5% in at least one population. The wild chicken population (AvDiv101) had the highest percentage of polymorphic markers (70%). The dam broiler line A3 was a close second with slightly less than 70% of SNPs polymorphic. The white egg layer line was clearly the population with the smallest number of polymorphic markers with less than 35% of SNPs informative (Additional File 2).

Heterozygosity was lower (P < 0.005) for the macrochromosomes (0.193) compared to the microchromosomes (0.207) when analyzed across all populations. Within most populations, heterozygosity was found to vary between chromosomal regions; for example, a three-fold difference was observed between GGA26 and GGA27 in the white egg layer population (Table 1).

Of the SNPs genotyped in Ceylon Jungle Fowl, 729 (90%) were successfully genotyped and 669 (92%) were not polymorphic in the sample of seven individuals. The alleles present in Ceylon Jungle Fowl were putatively inferred as the ancestral allele in *G. gallus*. Taken over all populations, ancestral frequencies were slightly lower in the microchromosomes (0.669 vs. 0.680 for macrochromosomes), but the difference was not significant. Population allele frequencies were skewed towards the ancestral state. The highest ancestral frequency was observed in the wild chicken population, and the lowest in the white egg layer and the Dutch breeds (Table 1, Additional File 3). Taken over all populations, ancestral frequencies were slightly lower in the microchromosomes (0.669, compared to 0.680 for macrochromosomes), but the difference was not significant.

### Linkage disequilibrium

Across all populations, LD for the microchromosomes was significantly lower compared to the macrochromosomes (P < 0.0001), and fitted values for LD were consistently lower for the microchromosomes for all populations. Differences in LD between micro- and macrochromosomes from the global fit to the Sved equation resulted in a 2.8× lower estimated recombination rate for the latter (Table 2). Observed values were almost consistently lower for the microchromosomes, although a few local exceptions were observed (e.g. dam broiler E5 showed somewhat higher LD at the microchromosomes at short distances).

**Table 2:** Estimated differences in recombination rate and Ne between micro- and macrochromosomes

Population	Rec._micro_/Rec._macro_	Ne_macro_	Ne_micro_	Ne_micro_/Ne_macro_
white egg layer	3.20	37.61	26.75	1.41
brown egg layer	4.14	160.31	147.34	1.09
sire broiler line	4.16	813.41	751.68	1.08
dam broiler E5	1.59	249.41	88.03	2.83
dam broiler A3	3.03	1097.47	738.9	1.49
Red Jungle Fowl	2.63	2473.92	1447.01	1.71
Owl Bearded	1.97	876.67	383.86	2.28
Frisian Fowl	3.96	634.39	558.06	1.14

*average*	2.76			*1.63*

Differences in recombination rate (Rec.) and effective population size (Ne) between micro and macro chromosomes estimated from fitting LD to the Sved curve. Differences in recombination rates were estimated using same Ne for micro- and macrochromosomes. Conversely, differences in Ne were based on a recombination rate 4.5 times higher in microchromosomes compared to macrochromosomes. This table shows the inconsistencies resulting from sampling different ranges of c while assuming that population sizes are static across generations (assuming that number of generations is 1/(2 c), c in Morgan [22]. See Additional File 4)

**Figure 1:**
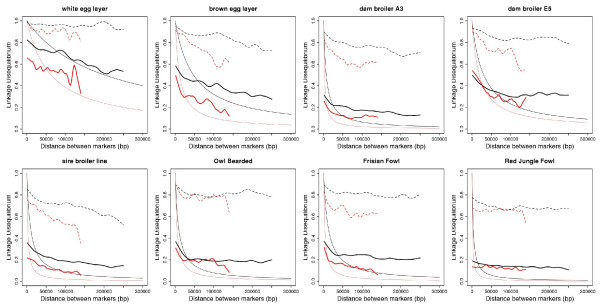
**Fitted and observed values of LD versus physical distance (bp), for the macrochromosomes (black) and microchromosomes (red)**. Observed values for r^2 ^are in thick lines (lowess fit through averages over a sliding window), Fitted values using the Sved equation [21] are thin continuous lines, and observed values for D' are in hatched lines (lowess fit through averages over a sliding window).

Observed values for r^2 ^were never < 0.1 even at distances of ~1 cM, contrary to the fit to the Sved equation predicted, and the r^2 ^values never reached high values (> 0.9) even at very small marker distances. This resulted in a much flatter observed versus predicted LD curves. Observed values for D' showed similar trends as r^2^, but were always much higher. Average D' never was < 0.5 at the macrochromosomes for any population even at 250 kb (Figure 1).

The white egg layer showed the highest LD of all populations, followed by the brown layer and the dam broiler line E5. The wild chicken had, in general, the lowest extent of LD, closely followed by dam broiler line A3 and the sire broiler line. The differences between the breeds were expected to depend on their effective population sizes, which were estimated to be between 40 and 1200 for the domesticated chicken populations, and > 2000 for the wild chicken population, based on all the macrochromosomes and estimated across all marker distances. Based on microchromosomes, Ne was estimated systematically lower for all populations, on average 1.6 times lower (Table 2). When marker distance was taken into account to allow estimation of Ne for a given point in the population history, a continuous reduction in Ne was observed for all populations (Additional File 4).

### Haploblock structure

**Figure 2:**
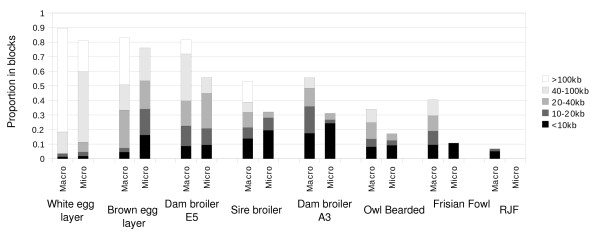
**Proportion of macro- and microchromosomes captured in haploblocks of different size**. Block defenitions were according to Gabriel et al. [10]. For a similar analysis based on the 4 Gamete Rule see Additional File 4.

The proportion of the regions captured by haploblocks was consistently lower for the microchromosomes compared to the macrochromosomes (P < 0.001, sign test). Haploblock sizes also tended to be smaller for the microchromosomes (Figure 2). Only in layers were more than 10% of the SNPs on the microchromosomes captured in blocks > 40 kb. Congruence in haploblock boundaries - both for micro- and macrochromosomes - between populations was very limited (Additional File 5).

Haploblock structure varied widely between populations (Figure 2). The white egg layer had large (> 100 kb) blocks covering 71% of the regions. In the brown egg layer and dam broiler line E5, block structure was still considerable with well over 40% of regions in blocks larger than 10 kb. There were pronounced differences between the results from different methods of block inference. In general, the Gabriel method inferred far fewer blocks that tended to be larger compared to the 4 Gamete Rule (Additional File 6). Nevertheless, the overall observations on differences between populations, and between micro- and macrochromosomes were consistent between the two methods.

### Haplotype homozygosity and evidence for selective sweep

**Figure 3:**
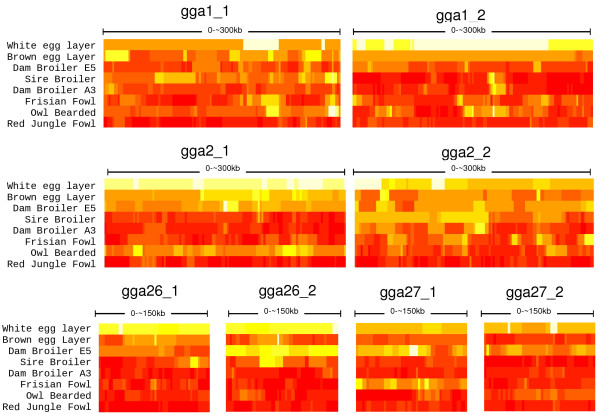
**Haplotype Homozygosity (HH) for all the populations and for all the genomic regions, sampled with bins of 10 SNPs along a sliding window**. High HH (1 haplotype present) is white, low HH is red. Lowest value of HH is 0.11, for Red Jungle Fowl. Intermediate values are shades of yellow and orange. Additional File 7 provides further insight in distribution of haplotypes.

Haplotype homozygosity (HH) measured over a sliding window with bin sizes of 10 SNPs (~20 kb) ranged from 0.11 to 1 (Figure 3). Over all populations, the microchromosomes showed consistently lower average HH, with the exception of dam broiler line E5. For the macrochromosomes a relatively small number (1-7) of haplotypes accounted for the vast majority of haplotype diversity (> 90%) in most domesticated populations while in the microchromosomes a larger number of haplotypes tended to explain a smaller part of the variation (Additional File 7). The white egg layer displayed extended regions with only a single haplotype, while HH was between 0.1 and 0.2 in Red Jungle Fowl for large parts of the regions covered in this study. All other populations showed a wide range of HH between and within regions.

### Haplotype sharing

**Figure 4:**
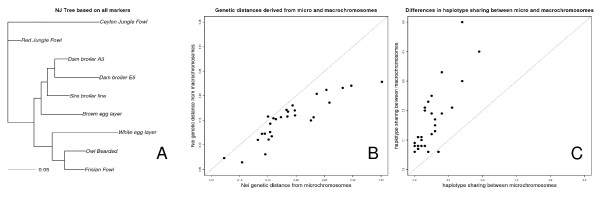
**A: Neighbor Joining tree based on genetic distances between population derived from all markers considered in this study**. **B:**Difference between haplotype sharing based on microchromosomal (horizontal axis) versus sharing based on macrochromosomal (vertical axis) haplotypes. Sharing was calculated as the average over a sliding window of window size of ~30 kb. Haplotype sharing is almost consistently lower in microchromosomes. **C: **Difference in genetic distances based on microchromosomal (horizontal axis) and macrochromosomal (vertical axis) genotypes. Genetic distances are almost consistently higher in microchromosomes.

Haplotype sharing on the microchromosomes was substantially lower (P < 0,0001, sign test) compared to the macrochromosomes (Figure 4C, Table 3), between 25 and 50% of sharing in macrochromosomes for haplotypes of the same physical size. Genetic distances between populations were also consistently larger (Figure 4B, P < 0.0001, sign test) based on genotypes derived from microchromosomal SNPs, but the difference was less pronounced compared to haplotype sharing.

**Table 3:** Haplotype sharing between populations.

Population	E2	B2	E3	E5	A3	AvDiv_101	AvDiv_9	AvDiv_10
white egg layer	-	0.07	0.06	0.00	0.05	0.00	0.05	0.04
brown egg layer	0.06	-	0.08	0.05	0.11	0.04	0.02	0.02
sire broiler line	0.15	0.19	-	0.08	0.19	0.00	0.03	0.03
dam broiler E5	0.09	0.19	0.33	-	0.14	0.01	0.01	0.01
dam Broiler A3	0.12	0.21	0.40	0.50	-	0.02	0.06	0.06
Red Jungle Fowl	0.06	0.06	0.08	0.07	0.08	-	0.03	0.01
Owl Bearded	0.25	0.10	0.20	0.11	0.13	0.08	-	0.14
Frisian Fowl	0.23	0.11	0.21	0.11	0.17	0.08	0.30	-

Haplotype sharing between populations. The table shows haplotype sharing based on a sliding window of 15 SNPs (~30 kb). Below each of the diagonals are the values for the macrochromosomes, above the diagonal are the values for the microchromosomes.

Haplotype sharing on the macrochromosomes was highest between the two dam broiler lines (Table 3). It was also high between the sire broiler line and the dam broiler lines, and between the two Dutch traditional breeds. The two Dutch traditional breeds exhibited haplotype sharing with the white egg layer. Patterns of sharing were consistent with genetic distances based on genotype data (Figure 4A).

## Discussion

Our aim was to study differences in LD and haplotype variation between micro- and macrochromosomes, using a very high marker density across populations reflecting commercial diversity as well as fancy breeds and wild chicken. Chromosomal regions were chosen to represent the maximum range in size of macro- (GGA1 and GGA2) and microchromosomes (GGA26 and GGA27); the two microchromosomes were among the smallest well-assembled chromosomes available within the current genome build [13]. Selection of SNPs was based solely on position (with the requirement of having 1 SNP every 2 kb), and thus systematic bias due to SNP selection was unlikely. Populations were chosen to reflect variation in the degree of polymorphism, and hence expected LD, to the widest possible extent, with the white egg layer at the lower end and Red Jungle Fowl at the upper end [18]. Extent of LD in chicken has been studied before [2,5,6] but these studies were limited in numbers of markers, marker density, population sampling or sampling across chromosomes to accurately and comprehensively asses LD to the same degree as the present study.

Based on higher recombination rates in microchromosomes compared to macrochromosome differences in LD and haplotype variation were expected, but measures of these differences have not been previously reported. We found LD, HH, haploblock structure, and haplotype sharing all consistently lower for microchromosomes compared to macrochromosomes when measured using physical distance. A direct effect of recombination on these measures comes from changing the relationship from physical distance to genetic distance. From the fit of LD to the Sved equation [21], and assuming that Ne is the same for all chromosomes, the recombination rate was estimated to be on average 2.8 times higher at the microchromosomes (Table 2). This difference is less than the expected 4.5 times higher recombination rate for microchromosomes compared to macrochromosomes [15]. The recombination frequency for the microchromosomes based on LD, therefore, appears to be systematically underestimated for all populations. While regional differences in recombination frequency are expected, currently no recombination map is available providing information at the scale of the present study (< 1 cM scale), not even for the macrochromosomes. For the smallest microchromosomes current recombination maps are even less detailed.

The inferred rate of 2.8× smaller recombination rate for macrochromosomes compared to microchromosomes, which is inconsistent with previous estimates (~4.5×, [15]), is due to a bias in the analysis from fitting the Sved equation across the same physical distance in micro- and macro chromosomes. LD at different distances has been shown to relate to effective population sizes at different numbers of generations by 1/(2 c), where c is the median distance between markers in Morgan [22]. By performing local fits to the data, using SNP distance bins that are similar in genetic rather than physical distances, the systematic difference in Ne between micro- and macrochromosomes disappears. For most populations, past population sizes derived from both classes of chromosomes become quite similar when measured against genetic distance (Additional File 4).

Since Ne does not seem to deviate systematically once distances are properly corrected for differences in recombination rate, the main explanation for observed differences in heterozygosity, genotype differentiation, and derived allele frequencies in the microchromosomes is higher mutation rate. Higher heterozygosity is known to be positively correlated to recombination rate [16,28], although the mechanism is not fully understood. We found derived allele frequency to be slightly higher on the microchromosomes, which suggests a higher evolutionary rate. A higher evolutionary rate for microchromosomes has been found before in a comparison between chicken and turkey macro- and microchromosomes [29]. Higher levels of differentiation could result from increased background directional selection for higher GC content in the microchromosomes. The effect of directional selection would have the same effect as a smaller effective population size. Since there is no evidence for differences in Ne a higher mutation rate seems to be the best explanation for higher genetic differentiation on the microchromosomes.

The Sved equation assumes a static population size [21]. However, the fact that the observed values or r^2 ^(Figure 1) show more of a flat line compared to the expected values of r^2 ^based on the fit to the global Sved equation is an indication of declining population size[22,24]. Fits based on local inter-marker distance-bins reveal declining effective population sizes as shown in Additional File 4. Differences in LD and derived effective population sizes are largely consistent with known population histories, with white egg layers known to be more inbred than other breeds, while most of the commercial broiler lines are considered outbred [18,30,17]. Nevertheless, the dam broiler E5 has been a closed line for many generations (AV, unpublished results), which explains higher LD and HH in this population. The decline of effective sizes for the eight populations is consistent with earlier findings of substantial loss of allelic variation in domesticated chicken [17], reemphasizing the concern to maintain genetic diversity in this species.

In humans, markers diagnostic for haplotypes, so called tag SNPs, are often transferable between populations because of haplotype sharing and populations having common haploblock boundaries [31]. In the chicken, haploblock boundaries show little overlap between populations, and haplotype sharing between populations is low. This difference between the two species could be the result of differences in demography, with the block-like structure of haplotype variation in humans being the result of population expansion in the past 10+ thousand of years originating from a population with an effective size of thousands to tens of thousands at most [23]. Conversely, the present study finds evidence for population contraction in chicken, which is consistent with the relatively small number of long haplotypes and levels of haplotype homozygosity. These long current haplotypes are expected to be a mosaic of a much higher diversity of small past haplotypes, similar to what is observed in dogs [3]. The ancient small haplotypes that make up today' s longer haplotypes, therefore, do not result in a very high r^2 ^(unless inbreeding becomes very high and only a very small number of haplotypes remain). They do, however, result in high D' as the inbreeding erodes away many of the possible - and previously existing - haplotypes in a population. D' will more often result in high LD values when only part of all possible haplotypes are present compared to r^2^[32,33]. Since the block construction methods applied here were based on *D' *it was not surprising to find considerable block structure - albeit often found in small blocks.

The block structure in the genomes of layers may be exploited to make genome-wide marker assays with 10,000 to 20,000 well chosen tag SNPs that would cover around 70% of the genome, supplemented by a similar number of SNPs to survey the remaining ~30%. Since block structure is mostly at the scale of < 10 kb for the more outbred broiler populations, and LD (measured as r^2^) near 0.2 at a similar scale, the number of informative SNPs would need to be > 100,000. However, as tag SNPs are probably not highly transferable between commercial populations, a general purpose assay might even need many more markers than 100 K.

Understanding sharing of haplotypes between populations is of further importance as it determines the success of transferring genetic parameters from one population to another [7]. The present study confirms the findings of Andreescu et al [5] in that high overlap in haplotypes between broilers exists. However, it appears to only exist between closely related populations. Transferability of marker information between more distantly related populations may be problematic. For the microchromosomes, haplotype sharing is very small even among the broilers, showing that population-to-population transferability of marker information should be treated differently for micro- and macrochromosomes at the same physical scale. Since genotype differentiation is also systematically higher for the microchromosomes, differences in haplotype sharing are likely the result of both increased mutation rate and recombination frequency for the microchromosomes.

## Conclusions

Patterns of LD, haplotype variation, and haplotype sharing, as well as genotype variation and genotype differentiation, are all different in the microchromosomes compared to macrochromosomes in chicken. While differences in LD are congruent with differences in recombination rate, differences in haplotype differentiation may be partly explained by an increased genotype differentiation. Differences in genotype differentiation seem best explained by a higher mutation rate for the microchromosomes. It is vital that whole-genome studies in chicken take these differences into account, both in the genotype assay design phase, as well as in interpretation and application of results. Because most birds have microchromosomes it is likely that the findings presented in this study are relevant to a wider group of avian species.

## Competing interests

The authors declare that they have no competing interests.

## Authors' contributions

HJM carried out genotyping and statistical analyses and drafted the manuscript. JB helped in interpreting results and drafting the manuscript. MG and RC coordinated the research. RC prepared DNA samples. RJ designed the marker assay. WM and HC helped in drafting and revising the manuscript. HK and AC aided in statistical analyses. RAV, OH, and PS provided DNA samples. All authors read, edited and approved the final manuscript.

## Supplementary Material

Additional file 1 An overview of the SNP selection. Two regions were sampled per chromosome, each region was ~1 cM in size. SNPs were selected with a spacing of ~2 kb. Coordinates of the regions are based on chicken genome build WASHU2. Click here for file

Additional file 2 Minor allele frequency (MAF) spectrum for the eight populations and Ceylon Jungle Fowl. Click here for file

Additional file 3 Ancestral allele frequency spectrum for the eight populations. Click here for file

Additional file 4 Estimates of past effective popution size. Estimates from microchromosomes were comparable to estimates from macrochromosomes. Click here for file

Additional file 5 Haploblock structure in the eight selected regions for eight populations derived from the Gabriel method [10]. Top and middle sections show two regions for GGA 1 and GGA 2 (macrochromosomes). Bottom section shows the four regions derived from the two microchromosomes (GGA26 and GGA27). 1 = white egg layer; 2 = brown egg layer; 3 = dam broiler E5 (closed line); 4 = sire broiler line; 5 = dam broiler A3 (open line); 6 = Frisian Fowl; 7 = Owl Bearded; 8 = Red Jungle Fowl. Click here for file

Additional file 6 Proportion of macro- and microchromosomes capture in haploblocks of different size. Block definitions were according to the 4 Gamete Rule [20]. Click here for file

Additional file 7 estimated number of major haplotypes (haplotypes occurring with a frequency > 5% in the populations studied). The second column for each of the populations shows the percentage of haplotype diversity explained by the major haplotypes. Click here for file
