# Supplementary material for: Comparison of linkage disequilibrium and haplotype diversity on macro- and microchromosomes in chicken
Source: BMC Genet. 2009 Dec 20;10:86. doi: 10.1186/1471-2156-10-86 (PMC2803787; doi:10.1186/1471-2156-10-86)
Supplement: Additional file 1 — An overview of the SNP selection. Two regions were sampled per chromosome, each region was ~1 cM in size. SNPs were selected with a spacing of ~2 kb. Coordinates of the regions are based on chicken genome build WASHU2. [file 1471-2156-10-86-S1.PDF]

Additional File 1: An overview of the SNP selection

chromosome	region	N snps	N worked	start	end	size
GGA1	1	152	144	87756012	88056143	300131
	2	148	139	135670843	135971996	301153
GGA2	1	148	136	14751544	15053410	301866
	2	152	135	43588767	43891658	302891
GGA26	1	77	66	2516476	2666304	149828
	2	72	67	3200535	3353106	152571
GGA27	1	72	62	1694035	1847001	152966
	2	68	57	2297912	2468857	170945

Two regions were sampled per chromosome, each region was ~1 cM in size. SNPs were selected with a spacing of ~2 kb. Coordinates of the regions are based on chicken genome build WASHU2.
