# Supplementary figures and images for: Comparison of linkage disequilibrium and haplotype diversity on macro- and microchromosomes in chicken
Source: BMC Genet. 2009 Dec 20;10:86. doi: 10.1186/1471-2156-10-86 (PMC2803787; doi:10.1186/1471-2156-10-86)

## Additional File 2

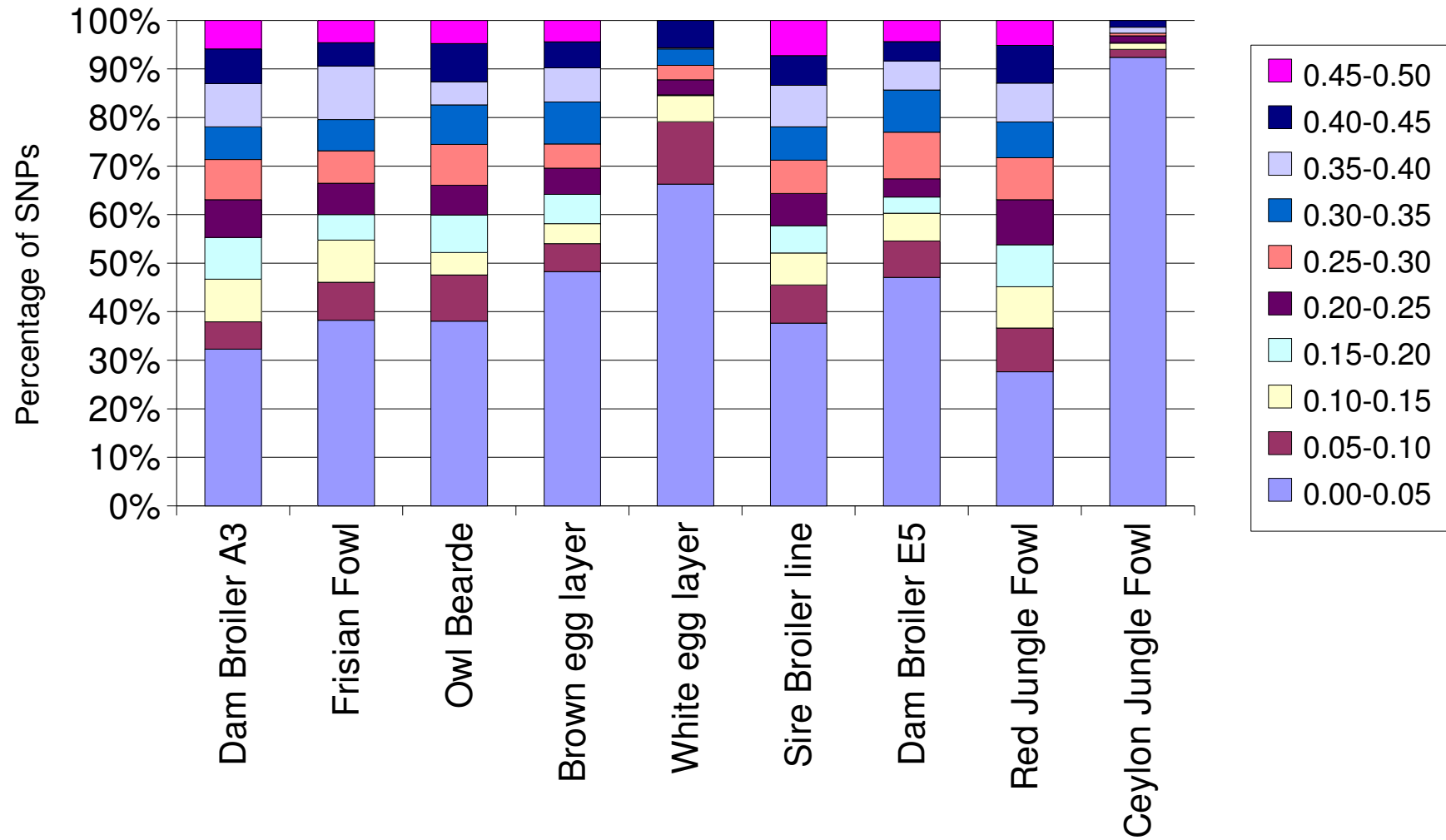

Supplement: Additional file 2 — Minor allele frequency (MAF) spectrum for the eight populations and Ceylon Jungle Fowl. [file 1471-2156-10-86-S2.PDF]

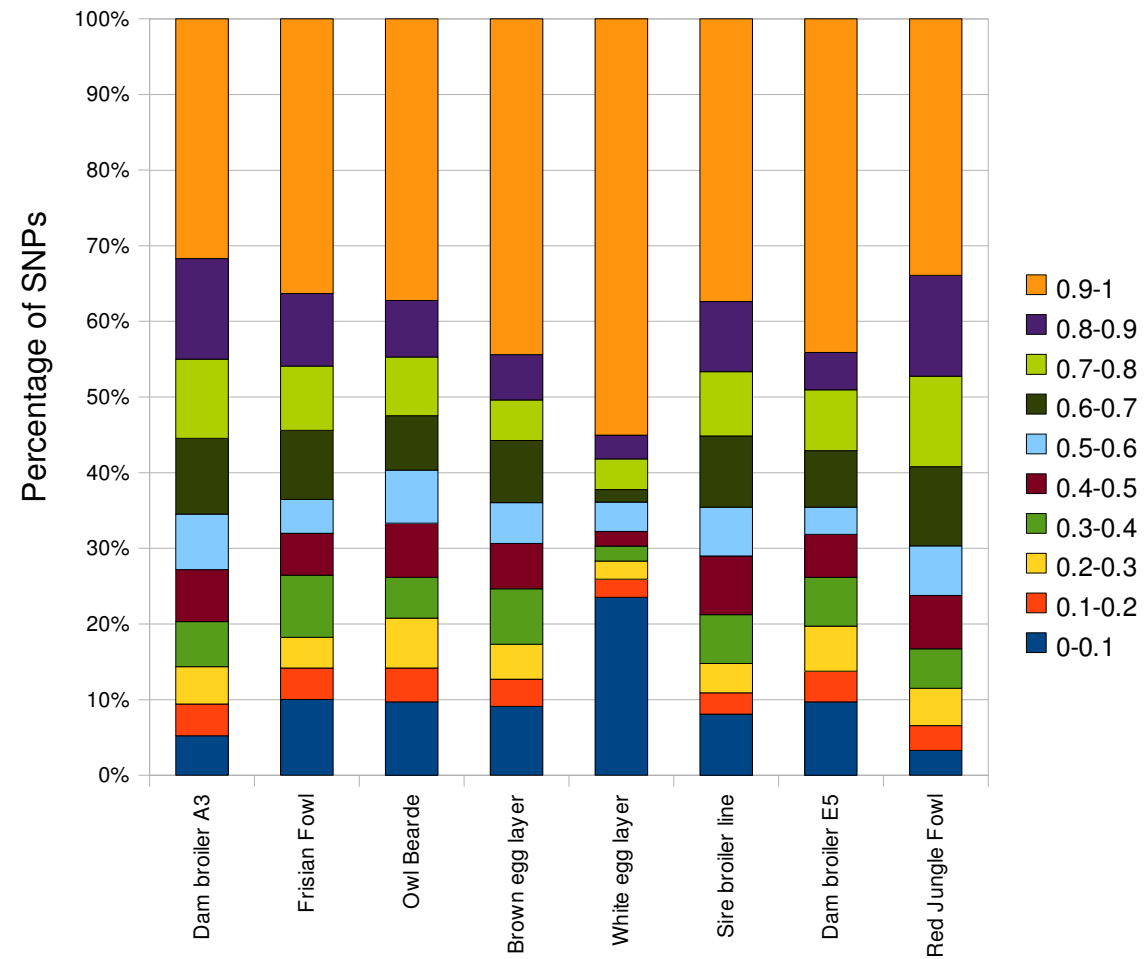

Supplement: Additional file 3 — Ancestral allele frequency spectrum for the eight populations. [file 1471-2156-10-86-S3.PDF]

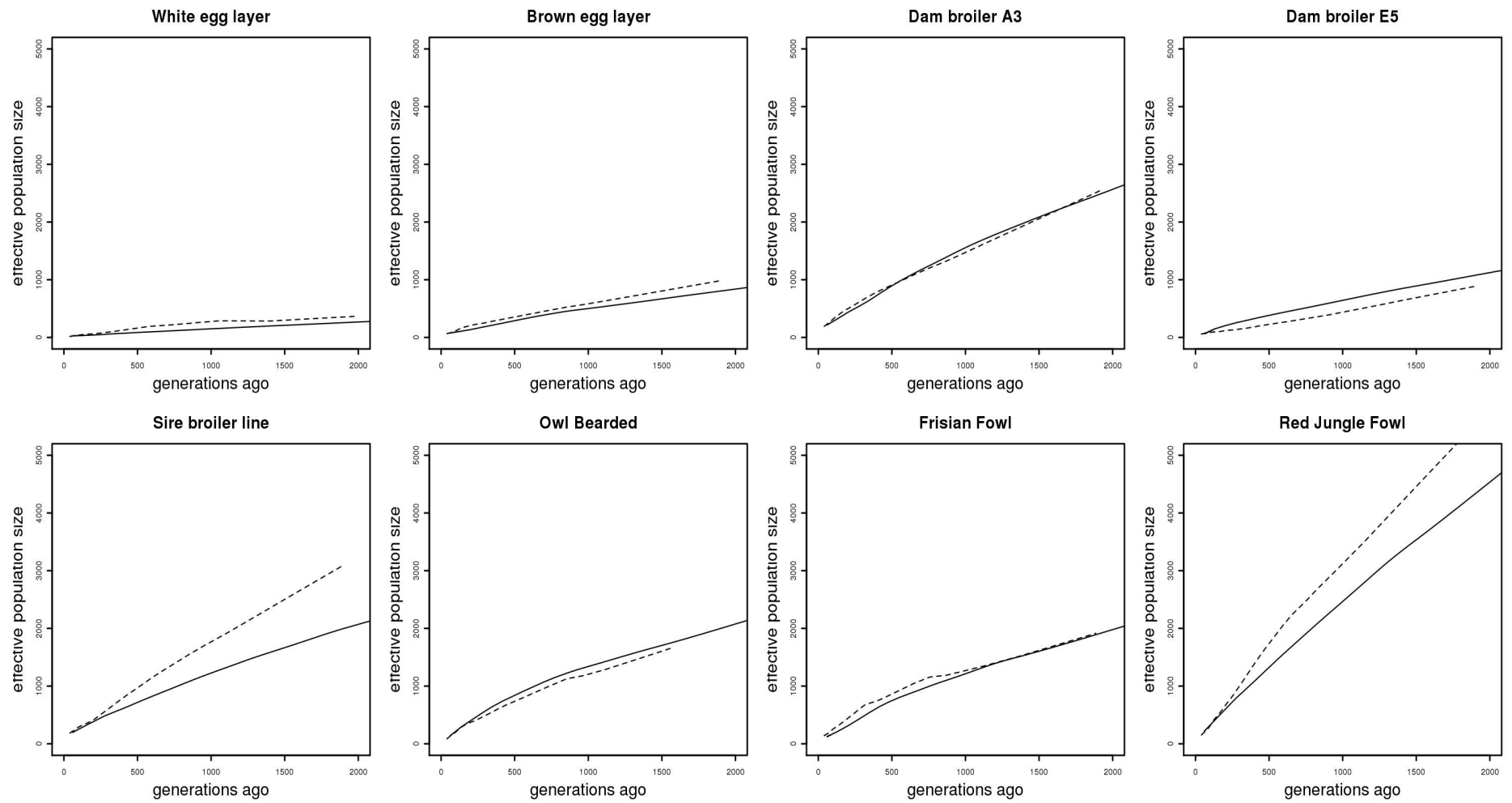

Additional File 4

Supplement: Additional file 4 — Estimates of past effective popution size. Estimates from microchromosomes were comparable to estimates from macrochromosomes. [file 1471-2156-10-86-S4.PDF]

Gabriel blocks, MAF>0.05

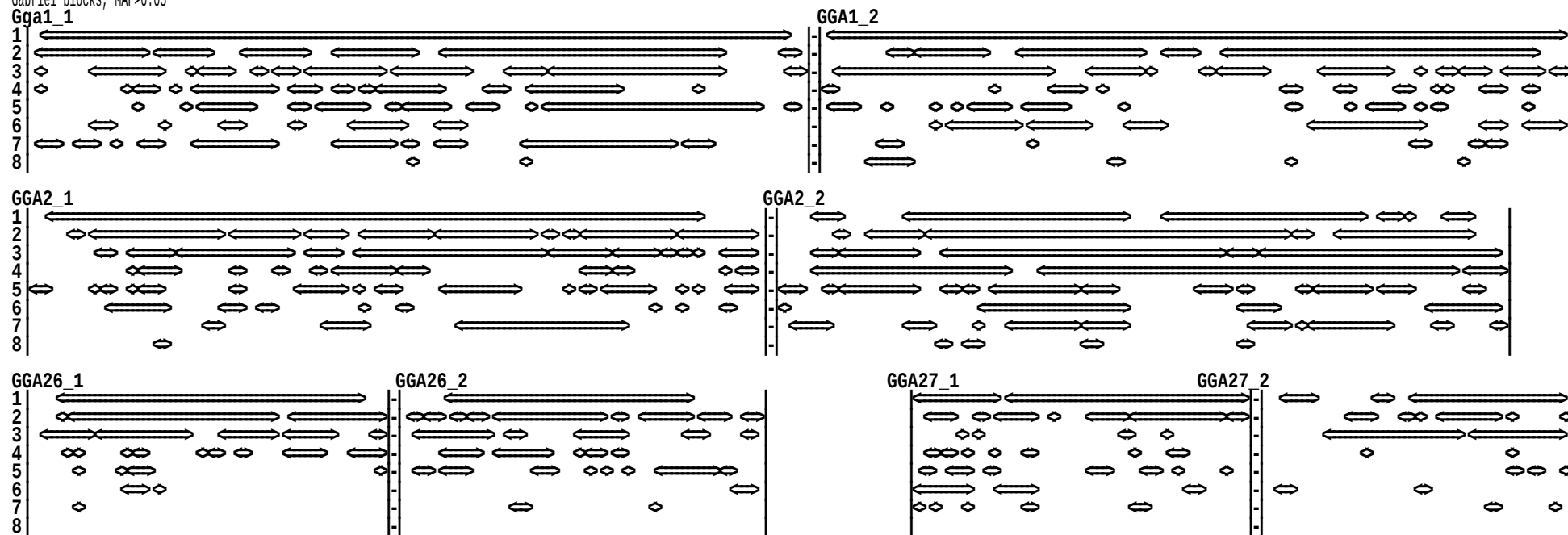

Additional File 5

Supplement: Additional file 5 — Haploblock structure in the eight selected regions for eight populations derived from the Gabriel method [10]. Top and middle sections show two regions for GGA 1 and GGA 2 (macrochromosomes). Bottom section shows the four regions derived from the two microchromosomes (GGA26 and GGA27). 1 = white egg layer; 2 = brown egg layer; 3 = dam broiler E5 (closed line); 4 = sire broiler line; 5 = dam broiler A3 (open line); 6 = Frisian Fowl; 7 = Owl Bearded; 8 = Red Jungle Fowl. [file 1471-2156-10-86-S5.PDF]

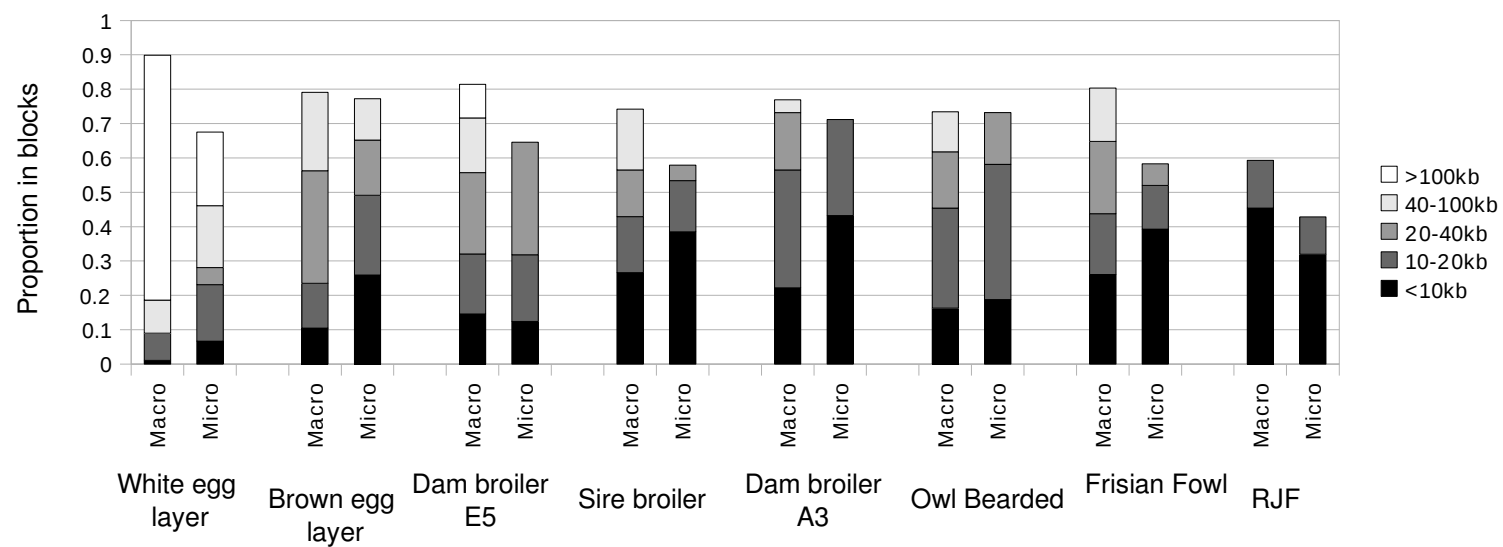

Additional File 6

Supplement: Additional file 6 — Proportion of macro- and microchromosomes capture in haploblocks of different size. Block definitions were according to the 4 Gamete Rule [20]. [file 1471-2156-10-86-S6.PDF]
