# Supplementary material for: Comparison of linkage disequilibrium and haplotype diversity on macro- and microchromosomes in chicken
Source: BMC Genet. 2009 Dec 20;10:86. doi: 10.1186/1471-2156-10-86 (PMC2803787; doi:10.1186/1471-2156-10-86)
Supplement: Additional file 7 — estimated number of major haplotypes (haplotypes occurring with a frequency > 5% in the populations studied). The second column for each of the populations shows the percentage of haplotype diversity explained by the major haplotypes. [file 1471-2156-10-86-S7.PDF]

Additional File 7: estimated number of major haplotypes

Haplocount-50

target-subtarget	all	E2		B2		E3		E5		A3		AvDiv_101		AvDiv_9		AvDiv_10		SRI	
	N	N	freq	N	freq	N	freq	N	freq	N	freq	N	freq	N	freq	N	freq	N	freq
gga1 region-1	40	2	0.99	4	0.88	3	0.76	5	0.97	5	0.84	3	0.47	8	0.96	4	0.87	4	0.93
gga1 region-2	39	3	1.00	2	0.91	3	0.46	5	0.98	7	0.91	5	0.74	5	0.90	7	0.87	4	1.00
gga2 region-1	35	1	0.92	3	0.94	6	0.95	4	0.95	7	0.84	4	0.54	7	0.83	4	0.87	6	1.00
gga2 region-2	43	2	0.91	3	0.94	2	0.89	4	0.93	7	0.98	6	0.76	7	0.89	9	0.79	6	0.93
gga26 region-1	50	2	0.95	5	0.83	5	0.63	4	0.88	8	0.78	6	0.57	6	0.67	7	0.67	5	1.00
gga26 region-2	56	2	0.95	10	0.85	5	0.73	4	0.93	8	0.72	6	0.64	9	0.83	4	0.69	6	1.00
gga27 region-1	46	3	0.92	4	0.88	5	0.68	3	0.82	4	0.62	6	0.79	6	1.00	5	0.52	6	0.92
gga27 region-2	45	3	0.85	8	0.81	3	0.24	3	0.85	6	0.50	3	0.50	6	0.58	8	0.81	5	1.00

Haplocount-25

target-subtarget	all	E2		B2		E3		E5		A3		AvDiv_101		AvDiv_9		AvDiv_10		SRI	
	N	N	freq	N	freq	N	freq	N	freq	N	freq	N	freq	N	freq	N	freq	N	freq
gga1 region-1	32	2	1.00	3	0.89	3	0.80	5	0.98	5	0.86	4	0.60	5	0.96	4	0.90	2	1.00
gga1 region-2	37	3	1.00	2	0.91	5	0.63	4	0.98	6	0.94	5	0.83	5	0.94	7	0.94	2	1.00
gga2 region-1	42	1	0.92	3	0.94	6	0.96	4	0.98	6	0.89	5	0.60	8	0.91	5	0.92	5	1.00
gga2 region-2	30	2	0.91	3	0.95	2	0.94	3	0.99	6	0.98	7	0.83	6	0.90	7	0.87	4	1.00
gga26 region-1	48	2	0.97	4	0.90	5	0.66	3	0.98	6	0.86	6	0.66	6	0.81	6	0.73	3	1.00
gga26 region-2	49	2	0.96	7	0.98	4	0.85	3	0.96	5	0.90	6	0.78	8	0.81	6	0.92	3	1.00
gga27 region-1	40	3	0.94	4	0.91	4	0.77	2	0.86	6	0.74	6	0.81	5	1.00	5	0.58	4	1.00
gga27 region-2	42	2	0.91	6	0.88	6	0.52	3	0.89	7	0.67	4	0.58	7	0.79	8	0.89	2	1.00

Estimated number of major haplotypes (haplotypes occurring in >5% among the studied populations). The second column for each of the populations shows the percentage of haplotypic diversity explained by the major haplotypes.
